# Stakeholder involvement in Multi-Criteria Decision Analysis

**DOI:** 10.1186/s12962-018-0120-0

**Published:** 2018-11-09

**Authors:** Praveen Thokala, Guruprasad Madhavan

**Affiliations:** 1 School of Health and Related Research, University of Sheffield, Sheffield, UK; 2 National Academies of Sciences Engineering, and Medicine, Washington, DC USA

**Keywords:** Stakeholders, Decision makers, MCDA, Priority setting

## Abstract

This brief perspective highlights the importance of decision maker buy-in and ownership through stakeholder engagement in the co-construction of the multi-criteria decision analysis (MCDA) model. A brief historical overview of MCDA is presented before outlining the importance of bridging the gap (and to gain trust) between the tool developers and users. The issues with the current MCDA tool development and testing efforts are highlighted, and the ownership and routine adoption of the MCDA process is discussed.

The past few years have witnessed a surge of interest in the use of multi criteria decision analysis (MCDA) for priority setting. Many different tools and techniques are available under the general heading of MCDA, and examples include the use of ‘Program budgeting and marginal analysis’ (PBMA) tools [1–3] for resource allocation decisions by local health care budget holders; use of discrete choice experiments to inform priority setting [4, 5]; use of the Evidem framework in making decisions about value for money of new health care technologies [6, 7]. However, the use of formal MCDA tools for health care priority setting is still limited, with most of the published MCDA studies being either pilots or hypothetical in nature [8]. This section outlines our reflections on why MCDA is not more widely used for priority setting and highlights key requirements for successful application of MCDA tools.

‘Buy-in’ from the key decision makers is imperative for the implementation of a structured priority setting process. In other words, if there is no buy-in from the decision makers, the results of the MCDA studies will not be implemented. Unfortunately, many of the MCDA studies seen in the literature on health care priority setting seem to ignore this fundamental requirement, with the MCDA researchers and practitioners working independently from the key stakeholders when conducting the MCDA. Examples of these include determining the criteria, performing the scoring and weighting independently from the stakeholders/decision makers. Whilst these studies may be methodologically sound, the outputs of the MCDA are not implemented as they are not trusted by the decision makers, given they had little or no involvement in the MCDA process.

There may be several potential reasons for the lack of ‘buy-in’ from the decision makers. One could be the reluctance to move away from cost-effectiveness analysis and its legacy, even if it does not include many other factors that affect real decisions [9]. This issue is not specific to MCDA; health care field has always been slow to adopt new techniques/methodologies [10, 11]. MCDA might be seen as too objective, transparent and explicit to allow the flexibility for making exceptions to individual cases. There may also be resistance to follow a process like MCDA as the decision makers may not want to reveal a set of priorities, over concerns that they may be challenged if the priorities shift over time. Furthermore, there could be other cultural/political factors that may affect the ‘buy-in’ from the decision makers.

The emphasis of MCDA researchers should be on educating the decision makers about the MCDA process and its benefits for supporting priority setting—for example, by conducting training courses or developing manuals to support MCDA process for priority setting. It should be noted that MCDA can help support the implementation of ‘accountability for reasonableness’ framework [12], put forward by Norman Daniels and other ethicists, which argues for the inclusion of due and fair process in setting and agreeing priorities.

‘Ownership’ of the MCDA process and results can help with achieving the ‘buy-in’ from the key decision makers/stakeholders. This involves engaging the stakeholders/decision makers in each step of the MCDA process [13], i.e. defining the decision problem, selecting the criteria, developing performance matrix, scoring alternatives and weighting criteria, aggregation, uncertainty analysis, and interpretation of results. The researchers should validate each step to ensure that MCDA design, input, and outputs are plausible and consistent with the decision maker objectives and stakeholder preferences [14]. Wherever needed, the researchers should help interpret the meaning of the different components of MCDA to ensure confirmation from the stakeholders.

The aim of this ‘co-production’ between the decision makers and MCDA researchers is to ensure that the stakeholders/decision makers feel that the decision is ‘their’ decision, not the tool’s decision. MCDA is a mechanism for making explicit the criteria and judgements that feed into the complex process of resource allocation. If the decision makers do not understand or feel like they were not of the process, they would be reluctant to use the MCDA results.

Many different tools and techniques are available under the general heading of MCDA. The technical complexity of MCDA, especially the weighted sum model, is very simple (each alternative’s scores on the criteria are multiplied by the weights and these weighted scores are then summed across the criteria to get a “total value” for each alternative) but the challenges are in the MCDA process (i.e. choosing the criteria, explaining the scoring/weighting to stakeholders, eliciting the preferences, presenting and interpreting the MCDA results). A key consideration is to ensure that the MCDA tools can support a committee or group of decision makers for use in real policy settings, as opposed to tools designed for single users [15, 16]. However, the MCDA tools cannot solve the problem on their own; they need engagement from the decision makers. Thus, rather than developing more tools, capacity building is more beneficial for the use of MCDA to support priority setting.

In summary, given MCDA is about eliciting the decision makers/stakeholders’ preferences, it is a fundamental limitation if they are not part of the whole MCDA process. The success of MCDA for priority setting relies on the buy-in and engagement from the health care bodies; and we should focus our efforts in supporting this rather than developing more MCDA tools.
